# The Effects of Topical Antiglaucoma Drugs as Monotherapy on the Ocular Surface: A Prospective Study

**DOI:** 10.1155/2014/460483

**Published:** 2014-06-09

**Authors:** Sevda Aydin Kurna, Semih Acikgoz, Ahmet Altun, Nurver Ozbay, Tomris Sengor, Osman Okan Olcaysu

**Affiliations:** ^1^Fatih Sultan Mehmet Education and Research Hospital, Ophthalmology Clinics, 34758 Istanbul, Turkey; ^2^Fatih Sultan Mehmet Education and Research Hospital, Pathology Clinics, 34758 Istanbul, Turkey; ^3^Bilim University, Florence Nightingale Hospital, Ophthalmology Clinics, 34724 Istanbul, Turkey; ^4^Erzurum Region Education and Research Hospital, Ophthalmology Clinics, 25000 Erzurum, Turkey

## Abstract

*Purpose*. The aim was to compare the effects of antiglaucoma eye drops on the tear functions and ocular surface. *Method*. Eighty-five eyes of 43 patients with glaucoma were included into this randomized prospective study. Timolol without preservative (1), timolol with benzododecinium bromide (2), latanoprost (3), bimatoprost (4), travoprost with benzalkonium chloride (5), and brimonidine with purite (6) were given to 6 groups. Schirmer I, tear film breakup time (TBUT), staining scores, and impression cytology samples were evaluated before and during 12-month-follow-up period. *Results*. At the end of 12 months, there was no detected change in Schirmer I and TBUT tests indicating dry eye. Corneal staining scores were higher in groups 1 and 2, while conjunctival staining scores were higher in group 6. Goblet cell count decreased in groups 1 and 5 in superior and inferior, group 2 in superior, and groups 3 and 6 in inferior conjunctiva. Squamous metaplasia grades showed a significant increase in groups 1 and 2 at 3rd, 6th, and 12th month controls (*P* < 0.05). *Conclusion*. We observed nonserious impact on tear functions and ocular surface with antiglaucoma monotherapy. Beta blockers induced more damage on the ocular surface suggesting the role of the dosing and active substances beside preservatives.

## 1. Introduction

Glaucoma is the second leading cause of blindness worldwide. Estimating a prevalence of 2.65% in the population over 40, the overall number of glaucomatous subjects is expected to increase in the course of the present decade, owing to both demographic expansion and population aging [1]. Topical hypotensive drops are the standard form of therapy, which is often used for long time in multiple dosing [2].

Experimental and clinical studies showed that the long-term use of topical drugs may induce ocular discomfort, tear film instability, conjunctiva inflammation, subconjunctival fibrosis, epithelial apoptosis, corneal surface impairment, and the potential risk of failure for further glaucoma surgery with a possible increase in visual loss [3, 4]. Ocular surface disease (OSD) demonstrates an overall prevalence of 42% (range 20–59%) in glaucoma, which is severe in 36% (range 14–66%) [5]. Associated symptoms are nonspecific to the anterior surface of the eye and may include dry eye, burning/stinging, itching, irritation, tearing, foreign body sensation, red eye, and blurred vision. Signs also generally are nonspecific and may include conjunctival staining with tear film abnormalities [6]. These side effects could be attributable to the active component as well as to the preservatives in the commercial medications but the mechanisms involved and the respective roles of the active compounds and the preservatives in inducing allergic, toxic, or proinflammatory effects of ophthalmic solutions are still being debated [7–10]. There are various commercially available antiglaucoma eye drops that a clinician must choose. Beside the clinical effectiveness, ocular surface changes that may affect patient's compliance should also be taken into consideration during this choice.

The purpose of this randomized and prospective study was to comparatively analyze the effects of various commercially available antiglaucoma eye drops as monotherapy on the tear functions and ocular surface for over the periods of 12 months.

## 2. Method

### 2.1. Subjects

Eighty-five eyes of 43 patients with the diagnosis of primary open angle glaucoma that had never used topical antiglaucoma drugs before enrolled in this study. The research was approved by the Ethics Committee and followed the tenets of the Declaration of Helsinki. Informed consent was obtained from the subjects.

Glaucoma was defined as intraocular pressure (IOP) more than 21 mmHg without treatment, abnormal automatic full threshold perimetry (30/2 Humphrey, San Leandro-Dublin, CA), and abnormal optic disc (increased vertical to horizontal cup to disc ratio, cup to disc asymmetry between the two eyes less than 0,2, and peripapillary splinter hemorrhages).

The exclusion criteria used were severe ocular trauma at any time, previous history of intraocular surgery or argon laser trabeculoplasty, current use of contact lenses, presence of eyelid or eyelash deformity, history of recent ocular inflammation or infection, previous or current use of other ocular medications including artificial tear therapy, systemic treatment known to affect tear secretion, autoimmune disease, and any history or slit-lamp evidence of eye surface disorders.

### 2.2. Groups

The patients were divided into six groups and started topical antiglaucoma drugs.

*Group 1 (8 patients, 15 eyes)*: 0,5% preservative-free timolol maleate twice a day. 

*Group 2 (7 patients, 14 eyes)*: 0,5% timolol maleate including 0,012% benzododecinium bromide (BDD) twice a day. 

*Group 3 (7 patients, 14 eyes)*: 0,005% latanoprost including 0,02% benzalkonium chloride (BAK) once a day at night. 

*Group 4 (7 patients, 14 eyes)*: 0,03% bimatoprost including 0,005% BAK once a day at night. 

*Group 5 (7 patients, 14 eyes)*: 0,004 travoprost including 0,015% BAK once a day at night.

*Group 6 (7 patients, 14 eyes)*: 0,1% brimonidine including purite 0.005% twice a day. 

### 2.3. Follow-Up

All patients received a complete eye examination including measurement of IOP by Goldmann applanation tonometry and viewing of iridocorneal angle and optic disc before the study. Conjunctival impression cytology was performed at 3rd, 6th, and 12th months for each eye. To evaluate the lacrimal function, tear film breakup time (TBUT), staining scores (SS), and Schirmer (SCH) I tests were performed before the study, at 1st week and at 1st, 3rd, 6th, and 12th months.

### 2.4. Tests & Studies

SCH I test with no topical anesthesia was performed using Schirmer's paper strip placed in the lateral lower conjunctival sac. The paper strip was removed after 5 minutes and the length of the moistened area was recorded.

Tear quality was measured with TBUT. To measure TBUT, a drop of sodium fluorescein dye was instilled and the average interval between the last complete blink and the appearance of first dry spot on the precorneal film was calculated under cobalt blue filtered light.

Corneal fluorescein staining was examined with cobalt blue illumination and lissamine green was then instilled and interpalpebral conjunctival staining of temporal and nasal conjunctiva was graded using the Oxford Scheme 6-point scale (from 0 to 5) [11].

Impression cytology was performed after one drop of topical anesthesia with the 4 × 5 × 6 mm sized rectangular shaped cellulose acetate filter papers of 0,025 *μ*m pore size (Millipore-GSWPO 4300) in superior-central and inferior-nasal bulbar conjunctiva. The specimens were stored in 95% ethanol and stained according to Papanicolau's modification of Gill's technique and periodic acid-Schiff. The specimens were examined under a light microscope in a masked fashion and were graded on a scale of zero to three according to Nelson's method. Goblet cell count was counted in 5 neighboring areas at ×400 magnification under light microscopy and mean goblet cell count of mm^2^ area was calculated. The same pathologist examined the specimens in a masked manner [12].

### 2.5. Statistical Analysis

All the data were analyzed using the NCSS 2007 software. Descriptive statistical methods (mean, standard deviation) were used for the demographic and clinical characteristics of cases. Variance analysis was used for the repeated measures of multiple groups; Newman Keuls post hoc multiple comparison test was used for subgroups analysis; one-way ANOVA test was used for the comparison of the groups and Tukey HDS test was used for the subgroups and Chi-square and Fisher tests were used for the comparison of the categorical variables. *P* values of <0.05 were considered statistically significant.

## 3. Results

Study included 85 eyes of 43 patients with the diagnosis of PAAG. Mean age of the patients was 50,67 ± 5,8 in group 1; 51,36 ± 5,5 in group 2; 55,57 ± 6,08 in group 3; 58,14 ± 8,69 in group 4; 57,43 ± 9,93 in group 5; and 58,33 ± 11,24 in group 6 (*P* = 0.09). Female to male ratio was 4/4, 4/3, 3/4, 4/3, 3/4, and 4/3 in groups 1, 2, 3, 4, 5, and 6, respectively (*P* = 0.84).

IOP significantly decreased in all of the groups at 1st week and at 1st, 3rd, 6th, and 12th months visits compared to baseline (*P* = 0.0001, *P* < 0.05).

### 3.1. Tear Functions

There was no statistically significant difference in mean SCH I test results between the groups at any time of the study (*P* > 0.05) (Table 1). There was no significant TBUT change in groups 1, 2, 3, and 6 before and after the treatment, while TBUT decreased significantly in group 4 at 1st week and 1st month controls (*P* = 0.048, *P* = 0.0019) and in group 5 at 1st week and 1st, 3rd, 6th, and 12th month controls (*P* = 0.018, *P* = 0.004; *P* < 0.05) (Table 1).

### 3.2. Staining Scores

There was no significant difference in corneal and temporal conjunctival SS in the baseline (*P* = 0.120; *P* > 0.05). Corneal SS were higher in groups 1 and 2 at 3rd, 6th, and 12th month controls (*P* = 0.04, *P* = 0.02; *P* < 0.05). Temporal SS showed significant increase to score 2 in group 6 at 12th month control (*P* = 0.011; *P* < 0.05). Nasal conjunctival staining was higher in group 4 at baseline (*P* = 0.06, *P* = 0.056; *P* < 0.05). Significant difference was observed between the groups at 1st, 3rd, and 12th month controls (*P* = 0.001, *P* = 0.028, *P* = 0.026; *P* < 0.05). Nasal SS were higher (score 2) in group 1 at 1st month, in groups 1 and 2 (score 2) at 3rd month and in group 2 (score 3) at 6th month, and in groups 4 (4 eyes) and 1 (1 eye) (score 2) at 12th month controls.

### 3.3. Impression Cytology

During impression cytology in the superior quadrant, squamous metaplasia grades showed significant differences between the groups in the 3rd, 6th, and 12th month controls. Groups 1 and 2 showed higher-grade values (*P* < 0.05). During the follow-up, squamous metaplasia grades showed a significant increase in groups 1 and 2 at 3rd, 6th, and 12th month controls when compared to the beginning (*P* < 0.05). Highest grade recorded in the superior quadrant was grade 2, which was observed in two eyes in group 1 (13,3%) in superior-central conjunctiva (Table 2).

**Figure 1:**
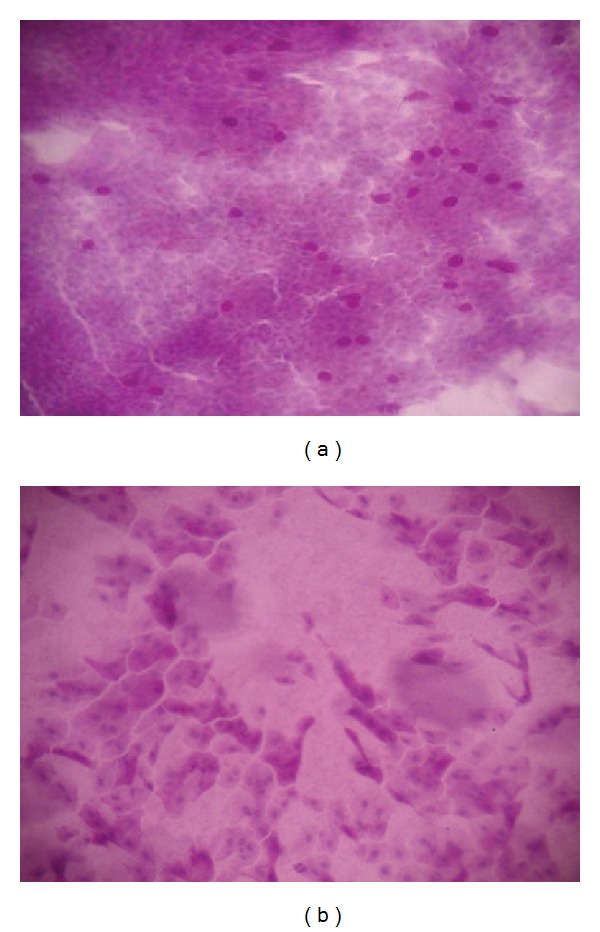
(a) The impression cytology of a patient in group 6 showing grade 0 metaplasia in the inferior-nasal conjunctiva before treatment. (b) The impression cytology of the same patient in group 6 showing grade 2 metaplasia in the inferior-nasal conjunctiva after using brimonidine-purite for 12 months.

In the inferior quadrant, squamous metaplasia grades showed a significant increase in groups 1 and 2 at 3rd, 6th, and 12th month controls when compared to the beginning (Table 3). However, group 6 showed a significant increase in squamous metaplasia grades in 3rd and 6th month control and group 5 showed a significant increase in squamous metaplasia grade only in 3rd month control when compared to the beginning. Highest grade recorded in the inferior quadrant was grade 2, which was observed in one each eye in groups 1 (6,7%) and 6 (7,1%) in inferior-nasal conjunctiva at 12th month control (Figures 1(a)-1(b)).

The count of goblet cells in superior-central conjunctiva showed a statistically significant difference at 3rd, 6th, and 12th month controls (*P* < 0.05). The mean count of goblet cells in superior-central conjunctiva in groups 1 and 2 at 3rd and 12th months and in group 5 at 12th month was significantly lower than beginning (*P* < 0.05) (Table 3). The mean count of goblet cells in superior-central conjunctiva in group 1 was significantly lower than groups 3, 5, and 6 at 3rd month control (*P* = 0.0215, *P* = 0.041; *P* < 0.05). The mean count of goblet cells in superior-central conjunctiva in groups 1 and 2 was significantly lower than group 3 at 6th month control (*P* = 0.029, *P* = 0.02; *P* < 0.05). The mean count of goblet cells in superior-central conjunctiva in group 1 was lower than groups 4 and 5 at 12th month control (*P* = 0.031, *P* = 0.032; *P* < 0.05).

There was statistically significant difference in count of goblet cells in inferior-nasal conjunctiva at 3rd, 6th, and 12th month controls (*P* < 0.05). The mean count of goblet cells in inferior-nasal conjunctiva in group 1 at 3rd, 6th, and 12th months, in groups 3 and 6 at 12th month, and in group 5 at 6th and 12th months was significantly lower than beginning (*P* < 0.05) (Table 4). The mean count of goblet cells in inferior-nasal conjunctiva in group 1 at 3rd month was significantly lower than groups 3, 4, 5, and 6 (*P* = 0.044, *P* = 0.005; *P* < 0.05). The mean count of goblet cells in inferior-nasal conjunctiva in group 1 at 6th month was significantly lower than groups 5 and 6 (*P* = 0.043, *P* = 0.036; *P* < 0.05). The mean count of goblet cells in inferior-nasal conjunctiva in group 1 at 12th month was significantly lower than groups 4, 5, and 6 (*P* = 0.016, *P* = 0.005; *P* < 0.05) (Table 4).

## 4. Discussion

Long-term use of topical medications in chronic ophthalmic conditions, such as glaucoma, may adversely affect the ocular surface [10]. However, the mechanisms of ocular surface damage as well as the respective role of the active compound and the preservatives in ophthalmic solutions are still being investigated [6, 10].

BAK is the most commonly used detergent preservative in topical ophthalmic preparations while BDD and oxidants such as stabilized oxychloro complex (SOC) or purite are other alternatives [4, 8–10]. BAK has been shown to cause tear film instability, loss of goblet cells, conjunctival squamous metaplasia, apoptosis, disruption of the corneal epithelium barrier, and damage to corneal nerves [13, 14]. Another alternative is BDD, which is also a quaternary ammonium surfactant, and may have properties similar to BAK. It is formulated with timolol as a gel-forming solution, which has a longer residence time [4]. Purite is an oxidative preservative which is usually used in brimonidine topical drops and artificial tears [15]. Clinical studies showed that purite caused the least amount of damage to corneal epithelial cells [9] and the number of inflammatory cells in the conjunctiva was significantly lower with brimonidine-purite [16]. Preservative-free drugs were less associated with ocular surface symptoms and signs in the literature [5, 17].

Active compounds as well as preservatives may affect the ocular surface. Beta blockers have been reported to inhibit proliferation of corneal epithelial cells [18] and lead to a decrease in goblet cell density and tear production [19, 20]. However prostaglandin analogues are claimed for inflammatory damage in the ocular surface of glaucoma patients combining allergy with toxicity [21, 22].

In the present study, we measured tear quality with TBUT and tear production with SCH I test and evaluated the ocular surface by corneal and interpalpebral conjunctival staining. We observed no statistically significant difference in mean SCH I test results between the groups at any time of the study or after. However, TBUT decreased significantly in groups 4 (bimatoprost) and 5 (travoprost) during their controls. None of the patients had Schirmer value less than 5 mm or TBUT value less than 10 mm indicating dry eye. The most significantly affected ocular sign of OSD was reported to be decreased TBUT, indicating tear film instability while corneal and conjunctival staining was a reliable indicator of severity in the literature [5]. Rossi et al. showed abnormal TBUT and punctuate keratitis, which was more frequent with increasing number of eye drops and number of instillations per day in the patients with topically treated glaucoma [23]. Shimazaki et al. [19] have reported in a prospective study that timolol caused significant impairments in tear production and turnover, while no adverse effects were observed on the corneal epithelial integrity and tear function with prostaglandin analogue unoprostone eye drops; Stewart et al. showed that timolol maleate demonstrated increased staining in the cornea and nasal conjunctiva from baseline to hour 0 and hour 1 on the healthy subjects [24]. Eyes being instilled with any type of beta blocker had more corneal epithelial punctate erosion and a shorter TBUT in a study by Lee et al. [18]. Kuppens et al. demonstrated that TBUT was significantly lower in patients treated with preserved and preservative-free timolol than in controls and did not differ significantly from each other, suggesting that the active compound may alter the tear film, while BAK may have other side effects [25]. These changes in tear function and tear turnover may increase both concentration and exposure time of drugs and preservatives. Furthermore, corneal anesthetic effect of timolol maleate may also be attributed to significant corneal toxicity in beta blocker instilling patients [20]. Supporting the findings of Inoue et al. and Stewart et al., we detected more corneal and nasal conjunctival staining in the patients in groups 1 and 2 (preserved and nonpreserved groups) receiving timolol suggesting the role of active compound timolol on corneal toxicity but on the opposite to Lee et al. and Kuppens et al., we did not detect a change in Schirmer I and TBUT tests with beta blocker eye drops.

Kozobolis et al. showed that central corneal mechanical sensitivity was reduced significantly in the patients receiving latanoprost, travoprost, and bimatoprost correlating with Schirmer and TBUT test scores [26]. However, Martone et al. have observed that clinical scores of corneal sensitivity, Schirmer I test, and lacrimal film breakup time were significantly lower in the preservative medication groups than in the preservative-free group with no significant difference between patients treated with timolol and latanoprost in the monotherapy group [10]. In our study, TBUT decreased significantly in patients using bimatoprost and travoprost monotherapy during their controls, while none of the patients using prostaglandin analogue showed significant staining over score 2. Tear functions were not affected in the patients using latanoprost although it represents a BAK concentration twice that of most other glaucoma drops.

Impression cytology is a simple and noninvasive method. Nelson et al. devised a 3-stage classification based on nuclear/cytoplasmic ratio and goblet cell density to examine conjunctival epithelium [27, 28]. The time required for onset of the metaplasia has been suggested to be less than three months [29]. Also in this study we detected squamous metaplasia mostly at 3rd month controls. Hong et al. evaluated the conjunctival changes in bulbar conjunctival impression cytology specimens from patients receiving timolol, latanoprost, dorzolamide, timolol + latanoprost, and timolol + dorzolamide medications according to Nelson's method. The impression cytology scores were significantly higher in the medication groups mostly in the fixed-combination therapy groups than in the monotherapy groups with no significant difference between the different types of medication after at least six months of treatment [30]. In the present study, squamous metaplasia grades did not show a significant difference between the groups at the end of 12th month controls in the inferior quadrant, while in the superior quadrant groups 1 and 2 showed higher-grade values (*P* < 0.05). Squamous metaplasia grades showed a significant increase only in groups 1 and 2 at 3rd, 6th, and 12th month controls when compared to the beginning in the superior and inferior quadrants. Beta blockers' exhibition of higher grade of squamous metaplasia might be related to active substance timolol and increased number of instillations per day in group 1 (nonpreserved timolol) and longer residence time of the gel-forming solution in group 2 (timolol + BBD).

The decrease in conjunctival goblet cell density is accepted as an important parameter in assessing the OSD. The conjunctival inflammation and reduced goblet cell density of dry eye are exacerbated by use of preserved topical agents [21, 31]. In our study, the mean count of goblet cells showed a significant decrease in groups 1 (nonpreserved timolol), 2 (timolol + BBD), and 5 (travoprost) in the superior quadrant while in the inferior quadrant in groups 1 (nonpreserved timolol), 3 (latanoprost), 5 (travoprost), and 6 (brimonidine) showed a decrease at the end of 12th month control. Baudouin et al. showed in the impression cytology specimens that class II antigen HLA-DR expression showed slight and nonsignificant increases in the glaucoma patients receiving a beta blocker as monotherapy or treated with a prostaglandin analogue alone (latanoprost, travoprost, and bimatoprost), while HLA-DR positivity was at the highest level in the multitreatment group [21]. Pisella et al. demonstrated that BAK-containing latanoprost and timolol exhibit higher inflammatory marker expression and decreased MUC5AC expression and proapoptotic effects on conjunctival cells than does nonpreserved timolol. Latanoprost caused less toxicity, however, than preserved timolol, and both drugs were less toxic than BAK alone in a study with flow cytometry in impression cytology specimens [32]. On the other hand, Noecker et al. found less damage in the cornea and lower inflammatory infiltrates in the conjunctiva with those drugs containing the least preservative concentrations, especially brimonidine-purite and bimatoprost using scanning electron microscopy and light microscopy in rabbits [33]. In the present study, we observed a decrease in goblet cell count in the timolol, latanoprost, and travoprost groups but not in bimatoprost group. Our observation of goblet cell decrease in these patients may reflect the role of inflammation accordingly.

This study has several limitations. Our number of patients for each group was limited and there was no control group for each active compound or preservative. Longer and larger scale prospective studies including control groups may be beneficial to improve glaucoma treatment and for a better understanding of ocular surface disease in glaucoma.

## 5. Conclusion

In this study, we observed nonserious impact on tear function tests and low-grade metaplasia with topical antiglaucoma monotherapy at the end of 12th month control. We also observed that preserved and nonpreserved beta blockers induce more damage on the ocular surface compared to prostaglandin analogues and brimonidine-purite suggesting that beside preservative substances, the number of daily administrations and active substances might also be responsible for the ocular surface changes observed.

## Figures and Tables

**Table 1:** SCH I (mm) values and tear breakup time (TBUT) of the tear film (second) according to the groups during the beginning, 1st week, 1st month, 3rd month, 6th month, and 12th month controls.

	Beginning	1st week	1st month	3rd month	6th month	12th month	*F*	*P*
SCH I (mm)								
Group 1	11.53 ± 4.45	13.73 ± 11.38	10.2 ± 5.48	11.67 ± 4.69	11.33 ± 4.97	11.27 ± 3.67	0.69	0.636
Group 2	12.86 ± 4.56	13.07 ± 3.52	12.93 ± 3.22	11.86 ± 2.18	11.29 ± 3.87	12 ± 3.01	0.81	0.547
Group 3	16.14 ± 7.03	13.71 ± 4.76	13.71 ± 5.47	13.14 ± 4.93	15.5 ± 4.97	14 ± 6.31	1.50	0.204
Group 4	10.93 ± 10.11	10.93 ± 8.53	9.79 ± 7.81	11.14 ± 8.38	11.29 ± 8.04	10.79 ± 8.19	0.47	0.796
Group 5	10.93 ± 3.17	9.43 ± 3.52	10.5 ± 3.23	8.79 ± 4.49	9.43 ± 3.88	9.14 ± 3.48	2.09	0.077
Group 6	11.86 ± 4.37	11.21 ± 5.58	11.21 ± 5.89	13.43 ± 4.65	12.5 ± 7.5	11.43 ± 7.15	0.75	0.592
*F*	1.50	0.92	1.21	1.88	2.22	2.27		
*P*	0.199	0.474	0.313	0.106	0.061	0.055		
BUT (sec)								
Group 1	14.57 ± 3.63	11.67 ± 4.39	10.27 ± 3.56	11 ± 4.31	11.47 ± 3.8	12.07 ± 3.88	1.04	0.402
Group 2	14.5 ± 4.99	12.21 ± 3.58	12.36 ± 3.97	10.21 ± 2.94	10.71 ± 2.3	10.21 ± 3.04	1.96	0.096
Group 3	18 ± 4.15	18.86 ± 2.51	18 ± 4.66	18.5 ± 2.07	18.64 ± 4.09	19.71 ± 2.87	0.73	0.602
Group 4	16.5 ± 4.03	14.14 ± 4.09	14.36 ± 4.47	15.36 ± 4.34	16.5 ± 4.7	18.29 ± 4.25	7.06	**0.001**
Group 5	19.29 ± 2.13	16.57 ± 3.32	15.64 ± 2.93	16.43 ± 2.34	15.64 ± 3.63	16.21 ± 2.99	5.76	**0.001**
Group 6	16 ± 2.94	16.71 ± 2.16	17.36 ± 2.76	16.57 ± 4.52	17.64 ± 4.67	18.21 ± 5.37	1.04	0.403
*F*	8.58	9.58	8.87	12.29	9.83	13.99		
*P*	**0.051**	**0.0001**	**0.0001**	**0.0001**	**0.0001**	**0.0001**		

**Table 2:** Squamous metaplasia grades in the superior conjunctiva according to the groups.

Squamous metaplasia Upper quadrant	Group 1	Group 2	Group 3	Group 4	Group 5	Group 6	*P*
Before	0.06 ± 0.25	0.14 ± 0.36	0.42 ± 0.51	0.14 ± 0.36	0.07 ± 0.26	0.35 ± 0.49	*χ*²: 10.29 *P* = 0.067
3rd month	0.73 ± 0.45	0.42 ± 0.64	0.42 ± 0.51	0.14 ± 0.36	0.28 ± 0.46	0.64 ± 0.63	*χ*²: 12.40 *P* = **0.030**
6th month	0.73 ± 0.45	0.85 ± 0.36	0.50 ± 0.51	0.07 ± 0.26	0.28 ± 0.46	0.35 ± 0.49	*χ*²: 23.91 *P* = **0.0001**
12th month	0.73 ± 0.70	0.64 ± 0.49	0.42 ± 0.51	0.07 ± 0.26	0.14 ± 0.36	0.42 ± 0.51	*χ*²: 19.58 *P* = **0.001**
*P*	**0.0001**	**0.0001**	0.392	0.194	0.061	**0.019**	
Before 3rd month	**0.002**	**0.046**	1.00	1.00	0.083	0.46	
Before 6th month	**0.002**	**0.002**	0.317	0.317	0.083	1.00	
Before 12th month	**0.004**	**0.008**	0.157	0.157	0.317	0.317	

**Table 3:** Squamous metaplasia grades in the inferior-nasal conjunctiva according to the groups.

Squamous metaplasia Inferior quadrant	Group 1	Group 2	Group 3	Group 4	Group 5	Group 6	*P*
Before	0.16 ± 0.25	0.21 ± 0.42	0.42 ± 0.51	0.28 ± 0.46	0.11 ± 0.26	0.28 ± 0.46	*χ*²: 7.92 *P* = 0.161
3rd month	0.53 ± 0.51	0.50 ± 0.65	0.57 ± 0.51	0.21 ± 0.42	0.35 ± 0.49	0.57 ± 0.75	*χ*²: 4.50 *P* = 0.479
6th month	0.40 ± 0.50	0.64 ± 0.49	0.57 ± 0.51	0.21 ± 0.42	0.28 ± 0.46	0.57 ± 0.75	*χ*²: 7.29 *P* = 0.200
12th month	0.53 ± 0.63	0.57 ± 0.51	0.71 ± 0.46	0.21 ± 0.42	0.28 ± 0.46	0.50 ± 0.65	*χ*²: 8.87 *P* = 0.114
*P*	**0.001**	**0.013**	0.096	0.392	**0.029**	**0.019**	
Before 3rd month	**0.008**	**0.046**	0.157	0.317	**0.046**	**0.046**	
Before 6th month	**0.025**	**0.014**	0.157	0.317	0.083	**0.046**	
Before 12th month	**0.008**	**0.025**	0.083	0.317	0.083	0.083	

**Table 4:** The mean count of goblet cells in superior-central and inferior-nasal conjunctiva.

Goblet cell count	Beginning	3rd month	6th month	12th month	*F*	*P*
Group 1						
Superior	93.4 ± 48.95	86.47 ± 44.14	86.93 ± 53.62	79.93 ± 42.75	5.68	**0.002**
Inferior	94.13 ± 48.67	87.27 ± 43.48	88.33 ± 54.6	81.47 ± 47.42	6.62	**0.001**
Group 2						
Superior	110.07 ± 41.49	99.21 ± 35.55	83.79 ± 43.54	85 ± 41.95	11.12	**0.001**
Inferior	120.43 ± 39.75	116.36 ± 35.09	113.29 ± 39.44	115.93 ± 34.18	0.61	0.616
Group 3						
Superior	128.57 ± 34.23	129.93 ± 27.59	136.71 ± 37.15	114.79 ± 24.27	2.21	0.103
Inferior	132.86 ± 25.77	131.64 ± 29.52	127.57 ± 35.44	113.5 ± 39.96	2.96	**0.044**
Group 4						
Superior	124.5 ± 30.58	105.5 ± 45.07	115.71 ± 38.79	119 ± 29.95	2.13	0.112
Inferior	132.21 ± 46.09	129.5 ± 42.33	126.21 ± 45.24	133.14 ± 35.97	0.33	0.802
Group 5						
Superior	132.86 ± 20.78	127.43 ± 19.57	125.5 ± 15.77	119.14 ± 15.04	4.36	**0.01**
Inferior	148.86 ± 19.71	140.14 ± 17.94	134.79 ± 19.97	128.14 ± 17.95	10.06	**0.047**
Group 6						
Superior	119.93 ± 41.51	130.79 ± 40.93	127.21 ± 55.32	114.21 ± 40.28	1.56	0.216
Inferior	174.07 ± 105.51	144.29 ± 51.59	135.64 ± 58.39	129.57 ± 43.14	2.89	**0.001**
*F*	2.17 3.43	3.75 4.32	3.81 2.40	3.98 3.75		
*P*	0.066 0.07	**0.004** **0.002**	**0.004** **0.044**	**0.003** **0.004**		

## References

[1] Quigley H, Broman AT (2006). The number of people with glaucoma worldwide in 2010 and 2020. *British Journal of Ophthalmology*.

[2] Kaštelan S, Tomić M, MetežSoldo K, Salopek-Rabatić J (2013). How ocular surface disease impacts the glaucoma treatment outcome. *BioMed Research International*.

[3] Fechtner RD, Godfrey DG, Budenz D, Stewart JA, Stewart WC, Jasek MC (2010). Prevalence of ocular surface complaints in patients with laucoma using topical intraocular pressure-lowering medications. *Cornea*.

[4] Baudouin C, Labbé A, Liang H, Pauly A, Brignole-Baudouin F (2010). Preservatives in eyedrops: the good, the bad and the ugly. *Progress in Retinal and Eye Research*.

[5] Baudouin C, Renard J-P, Nordmann J-P (2013). Prevalence and risk factors for ocular surface disease among patients treated over the long term for glaucoma or ocular hypertension. *European Journal of Ophthalmology*.

[6] Stewart WC, Stewart JA, Nelson LA (2011). Ocular surface disease in patients with ocular hypertension and glaucoma. *Current Eye Research*.

[7] Wilson LA (1996). To preserve or not to preserve, is that the question?. *British Journal of Ophthalmology*.

[8] Yee RW (2007). The effect of drop vehicle on the efficacy and side effects of topical glaucoma therapy: a review. *Current Opinion in Ophthalmology*.

[9] Noecker R (2001). Effects of common ophthalmic preservatives on ocular health. *Advances in Therapy*.

[10] Martone G, Frezzotti P, Tosi GM (2009). An in vivo confocal microscopy analysis of effects of topical antiglaucoma therapy with preservative on corneal innervation and morphology. *American Journal of Ophthalmology*.

[11] Sall K, Stevenson OD, Mundorf TK, Reis BL (2000). Two multicenter randomized studies of the efficacy and safety of cyclosporine ophthalmic emulsion in moderate to severe dry eye disease. *Ophthalmology*.

[12] Novack GD (1987). Ophthalmic beta-blockers since timolol. *Survey of Ophthalmology*.

[13] Baudouin C (2008). Detrimental effect of preservatives in eyedrops: implications for the treatment of glaucoma. *Acta Ophthalmologica*.

[14] Kahook MY, Noecker R (2008). Quantitative analysis of conjunctival goblet cells after chronic application of topical drops. *Advances in Therapy*.

[15] Katz LJ (2002). Twelve-month evaluation of brimonidine-Purite versus brimonidine in patients with glaucoma or ocular hypertension. *Journal of Glaucoma*.

[16] Noecker RJ, Herrygers LA, Anwaruddin R (2004). Corneal and conjunctival changes caused by commonly used glaucoma medications. *Cornea*.

[17] Pisella PJ, Lala E, Parier V, Brignole F, Baudouin C (2003). Effect of preservatives on the conjunctiva: a comparative study of beta-blocker eye drops with and without preservatives in glaucoma patients. *Journal Francais d’Ophtalmologie*.

[18] Lee S, Kim MK, Choi HJ, Wee WR, Kim DM (2013). Comparative cross-sectional analysis of the effects of topical antiglaucoma drugs on the ocular surface. *Advances in Therapy*.

[19] Shimazaki J, Hanada K, Yagi Y (2000). Changes in ocular surface caused by antiglaucomatous eyedrops: prospective, randomised study for the comparison of 0.5% timolol v 0.12% unoprostone. *British Journal of Ophthalmology*.

[20] Inoue K, Okugawa K, Kato S (2003). Ocular factors relevant to antiglaucomatous eye drop-related keratoepitheliopathy. *Journal of Glaucoma*.

[21] Baudouin C, Liang H, Hamard P (2008). The ocular surface of glaucoma patients treated over the long term expresses inflammatory markers related to both T-helper 1 and T-helper 2 pathways. *Ophthalmology*.

[22] Baudouin C (2005). Allergic reaction to topical eye drops. *Current Opinion in Allergy and Clinical Immunology*.

[23] Rossi GCM, Pasinetti GM, Scudeller L, Raimondi M, Lanteri S, Bianchi PE (2013). Risk factors to develop ocular surface disease in treated glaucoma or ocular hypertension patients. *European Journal of Ophthalmology*.

[24] Stewart WC, Stewart JA, Holmes KT, Leech JN (2000). Differences in ocular surface irritation between timolol hemihydrate and timolol maleate. *American Journal of Ophthalmology*.

[25] Kuppens EVMJ, de Jong CA, Stolwijk TR, de Keizer RJW, van Best JA (1995). Effect of timolol with and without preservative on the basal tear turnover in glaucoma. *British Journal of Ophthalmology*.

[26] Kozobolis VP, Detorakis ET, Maskaleris G (2005). Corneal sensitivity changes following the instillation of latanoprost, bimatoprost, and travoprost eyedrops. *American Journal of Ophthalmology*.

[27] Egbert PR, Lauber S, Maurice DM (1977). A simple conjunctival biopsy. *American Journal of Ophthalmology*.

[28] Nelson JD, Havener VR, Cameron JD (1983). Cellulose acetate impressions of the ocular surface. Dry eye states. *Archives of Ophthalmology*.

[29] Turaçli E, Budak K, Kaur A, Mizrak B, Ekinci C (1997). The effects of long-term topical glaucoma medication on conjunctival impression cytology. *International Ophthalmology*.

[30] Hong S, Lee CS, Seo KY, Seong GJ, Hong YJ (2006). Effects of topical antiglaucoma application on conjunctival impression cytology specimens. *American Journal of Ophthalmology*.

[31] Albietz JM, Bruce AS (2001). The conjunctival epithelium in dry eye subtypes: effect of preserved and non-preserved topical treatments. *Current Eye Research*.

[32] Pisella P-J, Debbasch C, Hamard P (2004). Conjunctival proinflammatory and proapoptic effects of latanoprost and preserved and unpreserved timolol: an ex vivo and in vivo study. *Investigative Ophthalmology and Visual Science*.

[33] Noecker RJ, Herrygers LA, Anwaruddin R (2004). Corneal and conjunctival changes caused by commonly used glaucoma medications. *Cornea*.

